# Biomass Derived Nitrogen-Doped Highly Porous Carbon Material with a Hierarchical Porous Structure for High-Performance Lithium/Sulfur Batteries

**DOI:** 10.3390/ma10101158

**Published:** 2017-10-06

**Authors:** Yan Zhao, Xiaomin Zhang, Yusen He, Ning Liu, Taizhe Tan, Chunyong Liang

**Affiliations:** 1 School of Materials Science & Engineering, Research Institute for Energy Equipment Materials, Tianjin Key Laboratory of Materials Laminating Fabrication and Interface Control Technology, Hebei University of Technology, Tianjin 300130, China; zhaoyan84830@hotmail.com (Y.Z.); zxm15510922072@163.com (X.Z.); hyshebut@163.com (Y.H.); 2 Synergy Innovation Institute of GDUT, Heyuan 517000, China; taizhetan@gdut.edu.cn

**Keywords:** nitrogen-doped, mesoporous, layered structure, hierarchical porous structure, lithium/sulfur batteries

## Abstract

A novel nitrogen doped mesoporous carbon (NMPC) with a hierarchical porous structure is prepared by simple carbonizing the green algae, which is applied as a host material to encapsulate sulfur for lithium/sulfur (Li/S) battery. The NMPC exhibits high pore volume as well as large specific surface area, and thus sulfur content in the S/NMPC composite reaches up to 63 wt %. When tested in a Li/S battery, the S/NMPC composite yields a high initial capacity of 1327 mAh·g^−1^ as well as 757 mAh·g^−1^ after 100 cycles at a current rate of 0.1 C, a reversible capacity of 642 was achieved even at 1 C. This good electrochemical performance of the S/NMPC composite could be attributed to a unique combination of mesopority and surface chemistry, allowing for the retention of the intermediate polysuflides within the carbon framework.

## 1. Introduction

As an attractive energy storage system, lithium/sulfur (Li/S) batteries have aroused wide concern in the past years due to its very high theoretical energy density (2600 Wh·kg^−1^) and specific capacity (1675 mAh·g^−1^), almost several times higher than that of the commercial lithium-ion batteries [1]. Therefore, Li/S batteries have been considered as one of the most promising candidates for the next-generation rechargeable batteries [2,3]. Nevertheless, some drawbacks have so far hindered its real-world applications, including the insulating of sulfur (5 × 10^−3^ S·cm^−1^ at 25 °C) and the shuttle loss of lithium polysulfides (Li_2_S_x_, 4 ≤ *x* ≤ 8) during cycling process [4].

To overcome the challenges described above, considerable efforts have been focused on developing the sulfur based composite cathodes. These sulfur based composite cathodes are prepared by incorporating sulfur into various conductive materials such as carbonaceous (e.g., porous carbon [5], carbon nanotube [6], and hollow carbon nanosphere [7]) and conductive polymer (e.g., polypyrrole [8], polyaniline [9] and polythiophene [10]) materials. Among them, porous carbons with high surface areas and good electrical conductivity are widely applied as sulfur hosts because they are beneficial for both enhancing the cathode conductivity and hindering the dissolution of polysulfides into the electrolytes [11]. Fabricating porous carbon-sulfur composite to improve the utilization of sulfur and cycling stability has been proven to be the most promising. The high electronic conductivity of porous carbon can conquer the electrical insulation of sulfur and its discharge products, and the large pore volume can provide enough room for volume change of sulfur cathodes during cycling [12,13]. For example, Wu et al. [1] used mushroom to encapsulate sulfur and increased the sulfur loading to 52 wt %, improving the electrochemical performance of lithium-sulfur batteries. Chen et al. [12] prepared a novel honeycomb-like nitrogen and oxygen dual-doped porous carbon/S composite, giving a high initial discharge capacity of 1185.4 mAh·g^−1^.

Additionally, previous reports have shown that the doped heteroatom (such as B, N, S, and O) in the porous carbon is a scheme to ameliorate the electrochemical performance for the carbon-sulfur cathode [14,15,16]. Among them, nitrogen has received growing attention, because it can induce chemical adsorption of sulfur which is beneficial to the cycling ability of porous sulfur/carbon cathodes [17,18]. Although the enhanced electrochemical performance has been realized in the recent studies for nitrogen-doped porous carbon/sulfur based composite cathodes, the preparation of nitrogen-doped porous carbon precursors has weaknesses in the cost and preparation conditions, weakening the possibility of commercial applications [19]. Consequently, exploiting more environmental friendly and efficient ways with inexpensive precursors are prerequisites for porous nitrogen doped carbon materials for industrial applications.

One efficient strategy is to design various nanostructured carbon with nitrogen doping derived from biomass materials, which as a precursor is economical and environmentally friendly [20,21,22,23]. For example, porous carbons derived from catkin [24], sisal [25], bagasse [26], corncob [27], and rice husk [28] have been used to composite with sulfur as potential cathodes for Li/S batteries.

Herein, we synthesize the novel nitrogen doped mesoporous carbon (NMPC) with hierarchical porous architecture derived from green algae to composite with S as cathode materials for high-performance Li/S batteries. The prepared NMPC shows a rich mesoporosity, which work as a polysulfide diffusion inhibitor for Li/S batteries. Moreover, the natural nitrogen doping can obviously improve the electron conductivity and strongly adsorb polysulfides, which will bring about a good rate capability and remarkable cycling stability for Li/S batteries. The effects of mesoporosity, hierarchical porous structure, and nitrogen doping on the electrochemical performance of the S/NMPC cathode are researched through a variety of characterization techniques.

## 2. Materials and Methods

The green algae were collected in Jin River (Tianjin, China) and NPMC was synthesized by simple carbonizing the green algae as the sole-source precursor for carbon and nitrogen. Firstly, algae was washed with DI water followed by dried at 60 °C overnight before use. The dried algae were carbonized at 800 °C for 2 h at a heating rate of 5 °C min^−1^ in Ar gas atmosphere, and then cooled to room temperature naturally, resulting in a black powder. Afterwards, sulfur (Shanghai Huzheng Nano, MS-P100, Shanghai, China) and the resulting NMPC powder were thoroughly mixed according to a 2.5:1 weight ratio of sulfur and carbon to yield a mixture. The S/NMPC composite was prepared by the heat treatment of the above mixture at 150 °C for 5 h under Ar flow, and the S content in the resulting product was around 63 wt %.

Characterization of the NMPC and S/NMPC composites were carried out by scanning electron microscopy (SEM, S-4800, Hitachi Limited, Tokyo, Japan) with 1 nm at 15 kV, X-ray diffraction (XRD, Rigaku-TTRIII, Tokyo, Japan), transmission electron microscopy (TEM, JEM-2100F, Tokyo, Japan), Raman spectra (LabRAM Hr800, HORIBA Jobin Yvon, Tokyo, Japan), Brunauer–Emmett–Teller (BET, ASAP 2020, Micromeritics, Norcross, GA, USA) analysis, X-ray photoelectron spectroscopy (XPS, VG ESCALAB MK II, VG Scientific, Princeton, NJ, USA), chemical analysis (CHNS, Vario Micro Cube, Elementar, Langenselbold, Germany), Fourier transform infrared spectroscopy (FTIR, Thermo Nicolet 6700, Waltham, MA, USA), respectively.

To evaluate the electrochemical performance of the S/NMPC composite, 2025-type coin cell was assembled in an Ar-filled glove box. The cell was composed of lithium metal as the counter- reference electrodes and the S/NMPC composite electrodes as the working electrodes separated by Celgard 2400 separator. The electrolyte was prepared by dissolving 1 M lithium bistrifluoromethanesulfonamide (LiTFSI) in tetraethylene glycol dimethyl ether as solvent with 0.1 M LiNO_3_ as an electrolyte additive.

The composite cathode slurry was composed of 80 wt % S/NMPC composite, 10 wt % PVDF, and 10 wt % acetylene black dissolved in 1-methyl-2-pyrrolidinone (NMP). The slurries were cast onto aluminum foil and then dried at 60 °C for 12 h. The electrodes were cut into small pellets of 1.5 cm in diameter with a punch, and the electrolyte amount is 75 μL. The specific capacities were calculated according to the mass of active sulfur, and the mass loading of active sulfur for each electrode was 3.5 mg·cm^−2^. The cyclic voltammogram (CV) measurements were performed from 1.0 to 3.0 V at a scanning rate of 0.1 mV·S^−1^ by using a PARSTAT 4000 electrochemical workstation. The electrochemical impedance spectroscopy (EIS) was conducted by a PARSTAT 4000 electrochemical workstation from 100 kHz to 10 mHz with an automatic scanning mode at room temperature. Galvanostatic charge/discharge curves were measured using on a multichannel battery tester (BTS-5V5mA, Neware) between 1 and 3 V vs. Li/Li^+^ electrode at different current densities. Applied currents and specific capacities were calculated on the basis of the weight of S in the cathode.

## 3. Results and Discussion

**Figure 1:**
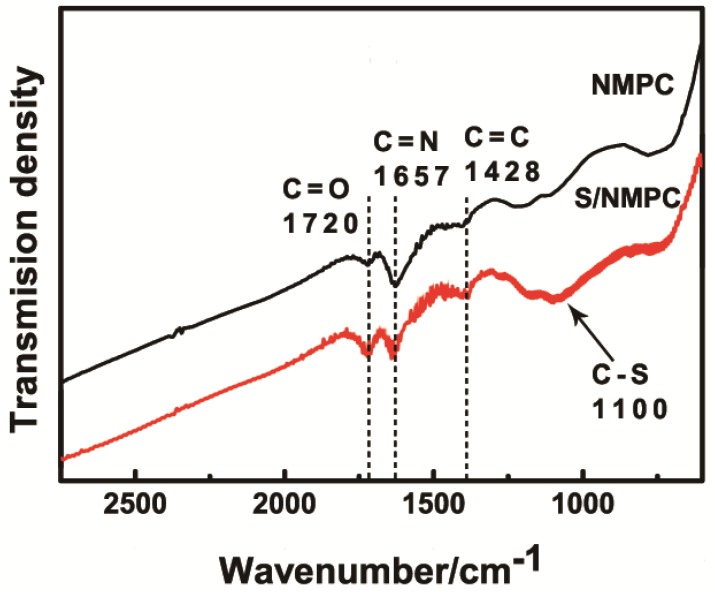
FTIR spectra of NMPC and S/NMPC composite.

Figure 1 presents the FTIR spectra of NMPC and S/NMPC composite sample over the range of 2750–600 cm^−1^. The C=O and C=N stretching vibrations are found at 1720 cm^−1^ and 1657 cm^−1^. It has been widely reported that nitrogen doping not only enhances the conductivity of the carbon material, but also captures polysulfides by increasing the interface adsorption properties of the carbon material [29,30,31]. The peak at 1428 cm^−1^ can be ascribed to the C=C stretching [10]. The characteristic peak at 1100 cm^−1^ can be ascribed to the C–S stretching compared to the sample NMPC, revealing the chemical bonding between elemental sulfur and NMPC [32,33].

**Figure 2:**
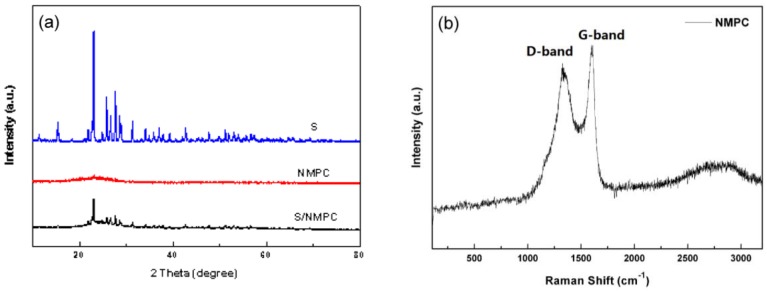
(**a**) XRD pattern of sulfur, NMPC and S/NMPC composite; (**b**) Raman spectra of as-prepared NMPC.

The XRD patterns of sulfur, as-prepared NMPC and S/NMPC composite samples are shown in Figure 2a. As-prepared NMPC displays a broad feature at 24° and a weak one at 43°, corresponding to the (002) and (100) planes, indicating that well-developed graphitic structures are dominant in the obtained NMPC. The XRD patterns of sulfur used in this work and S/NMPC composite exhibits Fddd orthorhombic structure for elemental sulfur. In comparison with S, the S/NMPC composite shows sharp peaks of S with reduced peak intensity. This results from the nanoscopic character of well-dispersed sulfur in the S/NMPC composite.

The Raman experiment was carried out to characterize the graphitization degree of the NMPC. As depicted in Figure 2b, two strong bands located at 1338 cm^−1^ and 1596 cm^−1^ are detected, corresponding to the D-band (sp3) and G-band (sp2) of carbon, respectively. In particular, the higher I_G_/I_D_ indicates the higher degree of graphitization.

**Figure 3:**
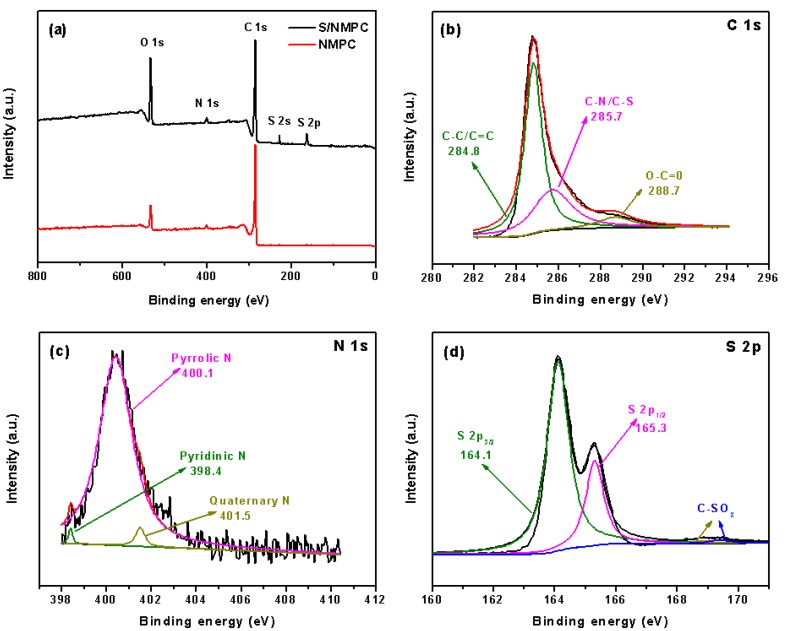
(**a**) Survey XPS spectrum of NMPC and S/NMPC composite; (**b**) C 1s; (**c**) N 1s; and (**d**) S 2p spectra of S/NMPC composite, respectively.

In order to further determine the surface chemical composition of the NMPC and S/NMPC composite, X-ray photoelectron spectroscopy (XPS) characterization was performed. Figure 3a shows the overall XPS spectrum of the NMPC, in which three typical characteristic peaks located at 287.5 eV, 401.9 eV, and 536.4 eV correspond to C 1s, N 1s, and O 1s, respectively, indicating that the NMPC mainly consists of C, N, and O elements, which indicates high purity of the samples. In the XPS spectrum of the C 1s (Figure 3b), the peaks centered at about 284.8 eV, 285.7 eV, and 288.7 eV corresponds to C–C/C=C, C–N/C–S, and O–C=O bonds, respectively [34]. Figure 3c reveals the high resolution peak of N 1s, which can be divided into three fitted peaks with different binding energy. The three component peaks are assigned to quaternary N (401.5 eV), pyrrolic N (400.4 eV), and pyridinic N (398.4 eV), respectively, suggesting that the nitrogen atoms originated from the algae are successfully introduced into the carbon lattice. As calculated by the XPS results, the NMPC has a nitrogen doping level of 1.45%. Among these types of nitrogen, the pyridinic N and pyrrolic N are more effective in forming LiSnLi^+^ N binding and adsorb polysulfides (Li_2_Sn) via the lone-pair electrons in nitrogen.

The high resolution S 2p peaks (Figure 3d) can be divided into four components including S 2p_3/2_ (164.1 eV), S 2p_1/2_ (165.3 eV), and C–SO_x_ bonds (168.8–169.4 eV) [35], revealing the sulfur dominated in the NMPC framework via the formation of C–S bond. These results suggest that sulfur is embedded in mesoporous structure after the heat treatment with chemical interaction with NMPC.

**Figure 4:**
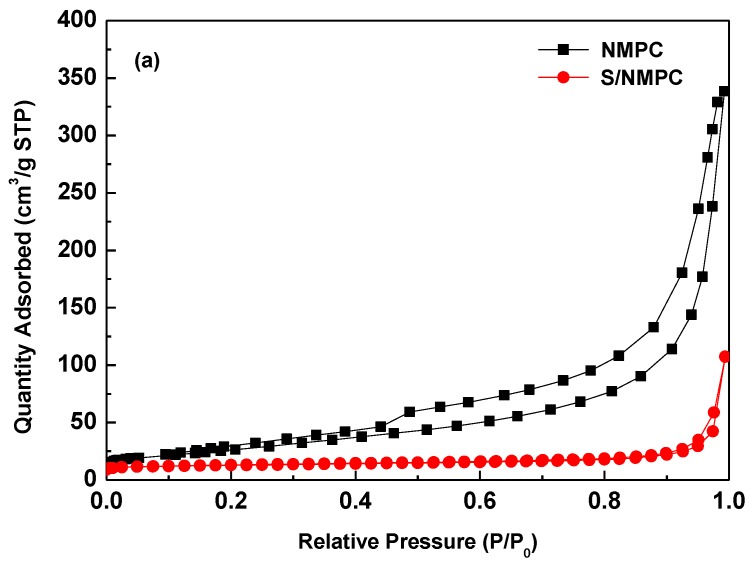

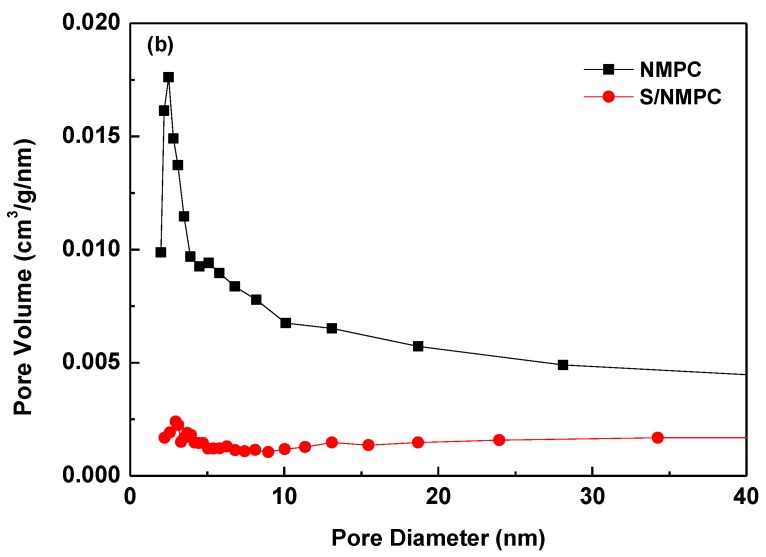
(**a**) N_2_ adsorption-desorption isotherms and (**b**) BJH pore size distribution of the NMPC and the S/NMPC composite.

The N_2_ adsorption/desorption isotherms and pore-size distributions profiles of the NMPC and the S/NMPC composite are shown in Figure 4a,b, respectively. The N_2_ adsorption/desorption isotherm of NMPC exhibits a type IV isotherm, indicating the existence of mesopores. Moreover, the pore size distribution for NMPC declares the existence of mesopores, ranging from 2 to 40 nm in size, and the pore size distribution (PSD) was calculated by the Barrett–Joyner–Halenda (BJH) method using the adsorption isotherm. For the NMPC, its BET surface area and pore volume are 101 m^2^ g^−1^ and 0.54 cm^3^ g^−1^. The high BET surface area can serve as channels for the rapid transport of ions to insulating S_8_ and Li_2_S_2_/Li_2_S precipitates. After heat treatment, the specific surface area and pore volume of the S/NMPC has decreased significantly to 16 m^2^·g^−1^ and 0.16 cm^3^·g^−1^. Therefore, it is believed that sulfur has penetrated into the multiple pore structure and filled the mesoporous of the NMPC.

**Figure 5:**
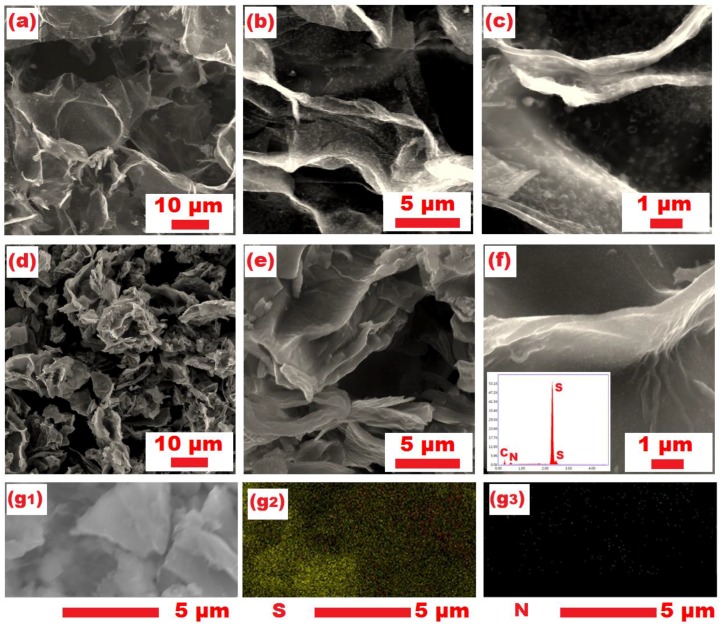
Typical SEM images of (**a**–**c**) NMPC and (**d**–**f**) S/NMPC composites; (**g1**) SEM image of a selected region of the S/NMPC composite and (**g2**, **g3**) the element mapping of sample S/NMPC.

Figure 5a–f shows SEM images of the NMPC and S/NMPC composite. The NMPC clearly displays a hierarchical porous structure with interconnected 3D pores (Figure 5a–c). This suggests that the NMPC not only can effectively improve electron transport, but also can accommodate the volume change during discharge/charge process. After sulfur loading, it can be observed that the S/NMPC composite remains a hierarchical porous structure (Figure 5d–f). Results of EDX mapping for nitrogen, sulfur, and carbon elements are also shown in Figure 5g. The bright regions represent these elements distributed in the composite, and indicate the sulfur and nitrogen could be homogeneously distributed within the nanopores of NMPC. The EDX spectrum analysis shows the material to contain C, N, and S and the graph is shown in Figure 5f (inset).

**Figure 6:**
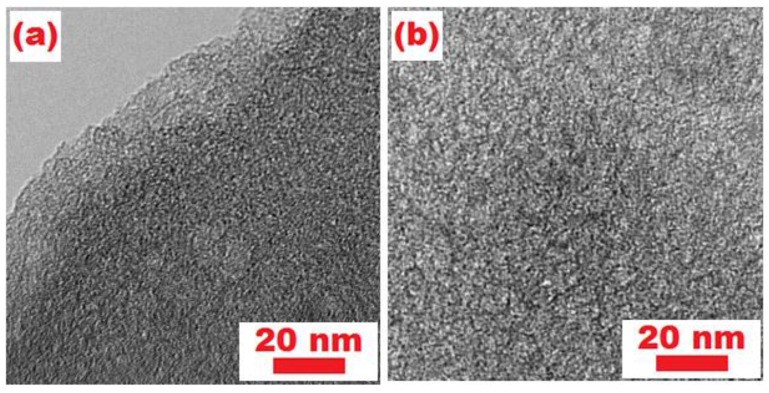
TEM images of sample (**a**) S/NMPC and (**b**) NMPC.

Figure 6a–b shows the morphology and microstructure of the S/NMPC and NMPC. A great number of white spots are clearly found from high magnification TEM (Figure 6b), which represents the formation of numerous pores. As a result, the NMPC material exhibits a large specific surface area and high porosity with interconnected 3D nanopores. However, only a few white spots can be found in the S/NMPC composite from Figure 6a, indicating that the sulfur has been completely encapsulated into the nanopores of the NMPC.

**Figure 7:**
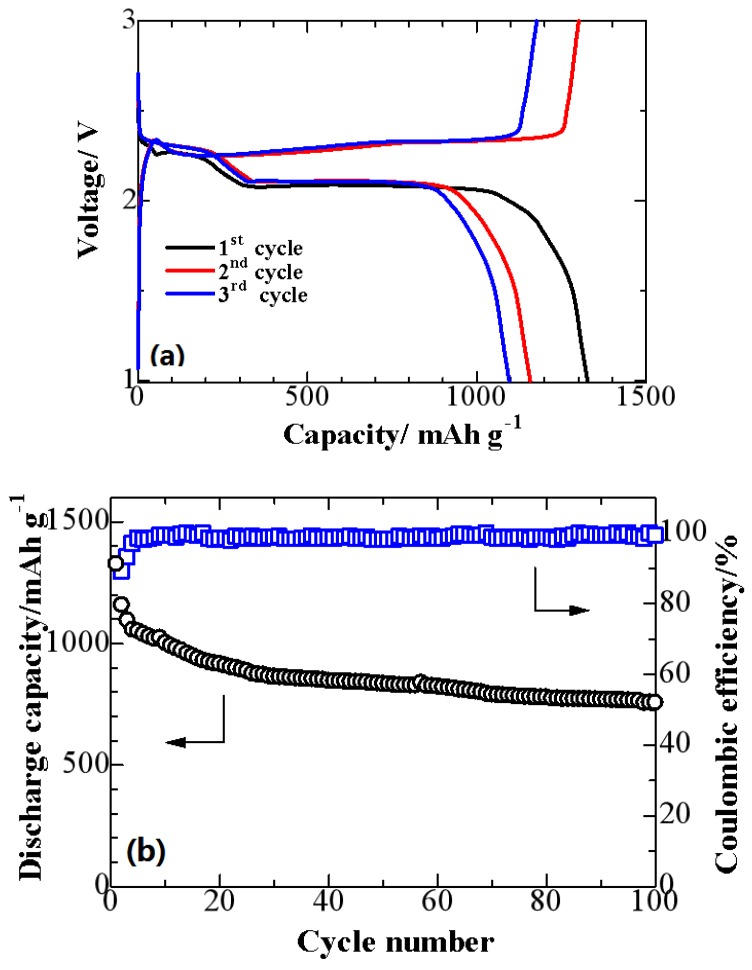
(**a**) Discharge/charge performance of a S/NMPC/Li cell at 25 °C at 0.1 C between 1 V and 3 V and (**b**) cycling performance and coulombic efficiency of a S/NMPC/Li cell at 25 °C at 0.1 C.

The initial discharge/charge profiles and cycling performance of the S/NMPC composite at 0.1 C (C is the theoretical capacity of the battery expressed in amp-hours, and a battery with a capacity of 1 amp-hours should be able to continuously supply a current of 1 amp to a load for exactly 1 h, 0.1 amp for 10 h before becoming completely discharged and the applied current is 167.2 mA·g^-1^) are presented in Figure 7a,b, respectively. One can see in Figure 7a that the profiles show two voltage plateaus upon discharging and one voltage plateau upon charging, two-step reversible reaction of S↔S_n_^2−^ (*n* ≥ 4)↔S_2_^2^. The first short discharge plateau at about 2.3 V is associated with the oxidation of S to high order polysulfides (Li_2_S_n_, *n* ≥ 4). The discharge plateau around 2.0 V represents the decomposition of the polysulfide chain to produce insoluble lithium Li_2_S_2_ and Li_2_S [36,37]. Figure 7b displays the cyclability of the S/NMPC composites, which yields a high initial discharge capacity of 1327 mAh·g^−1^ at a current density of 0.1 C and shows good stability with 757 mAh·g^−1^ reversible capacity after 100 cycles, and the average coulombic efficiency of the cathode is about 98%. The enhanced electrochemical performance of S/NMPC composite is attributed to its porous structure, providing strong absorbability to retain polysulfides dissolved in the electrolyte.

**Figure 8:**
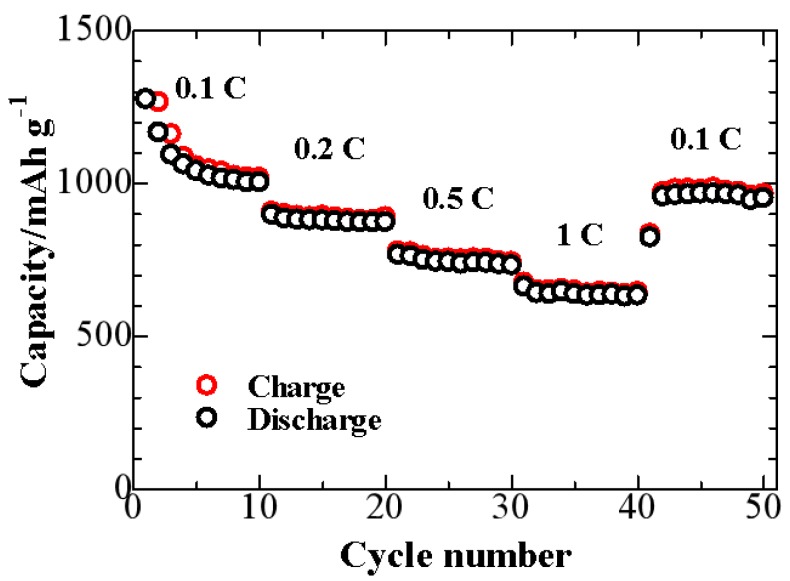
Rate performance of a S/NMPC/Li cell at 25 °C at various current densities.

The rate capability of S/NMPC composite is shown in Figure 8. The stable specific capacities of S/NMPC composite at 0.1 C, 0.2 C, 0.5 C, and 1 C are 1025 mAh·g^−1^, 877 mAh·g^−1^, 743 mAh·g^−1^, and 642 mAh·g^−1^, respectively. Furthermore, the discharge capacity of 967 mAh·g^−1^ can still be obtained when the current density returned back to 0.1 C, indicating that the S/NMPC composite is highly robust and stable.

**Figure 9:**
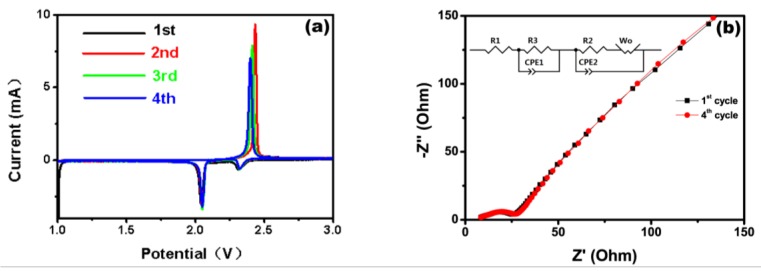
(**a**) CV curves of a S/NMPC/Li cell at 25 °C from first to fourth at a sweep rate of 0.1 mV·S^−1^; and (**b**) Electrochemical impedance spectra of a S/NMPC/Li cell at 25 °C after first and fourth discharge/charge cycle.

Cyclic voltammetry (CV) curves of Li/S cells with representative samples S/NMPC as cathode materials are shown in Figure 9a. Two well-defined reduction peaks at about 2.3 V and 2.0 V are assigned to the multistep reduction of elemental sulfur, which corresponds to reduction of element sulfur to soluble lithium polysulfides (Li_2_S_n_, *n* = 4–8) and further conversion of these lithium polysulfides to insoluble Li_2_S_n_ (*n* = 1, 2), respectively. The wide oxidation peak around 2.5 V is mainly attributed to the oxidation of Li_2_S_n_ (*n* = 1, 2) into high-order soluble polysulfides. Noticeably, there are only slight changes for CV peak of S/NMPC in subsequent cycles and in the subsequent scans, the oxidation peaks are shifted to lower potentials, indicating an improvement of reversibility of the cathode with cycling. This indicates that nitrogen doping plays an important role on suppressing the diffusion of lithium polysulfides and minimizing the mass loss of active materials during cycles.

Electrochemical impedance spectroscopy (EIS) was used to detect the interfacial resistance and lithium ion mobility resistance of the electrode. Figure 9b shows EIS of the S/NMPC electrode at the first and fourth cycles. The Nyquist plots for the electrode consist of a semi-circle in high frequency regions and an inclined line at low frequency region, which can be imputed to the contact resistance and ion diffusion resistance during the charge and discharge processes, respectively. The circuit includes electrolyte resistance R1, charge transfer resistance R3 and resistance of anode metallic Li R2. As cycle number increases from 1 to 4, the observed R1 slightly increased, suggesting that the heteroatoms of nitrogen have an outstanding interaction among various polysulfides, which leads to slight dissolution of various long-chain polysulphides in the electrolyte; and the increase in the R3 should be ascribed to the increased victory of electrolyte. Unlike R1 and R3, the observed R2 first decreases and then increases with cycle number increasing from 1 to 4. After four cycles, the porous materials are well activated so that Li^+^ ions can easily reach to the cathode and the abundant deposition of insulating solid products of Li_2_S_2_/Li_2_S on the anode metal Li increases the hardship for Li^+^ to cross out the solid products to react with sulfur cathode.

## 4. Conclusions

NMPC was synthesized by carbonizing the green algae and the S/NMPC composite has been prepared via a simple melt-diffusion technique. The unique combination of several merits of NMPC—including mesopority, hierarchical porous structure, and surface chemistry—renders this material especially suitable as a conductive matrix for strong polysulfides confinement. The hierarchical porous structured NMPC with a highly porous structure gives rise to a high sulfur loading and an effective suppression of polysulfides. Furthermore, the doping nitrogen in the carbon enhances electron conductivity and offers a strong chemical-absorption ability of polysulfides, which gives rise to the great electrochemical properties of S/NMPC composite as a cathode for lithium/sulfur batteries. At 0.1 C, the initial discharge capacity was up to 1327 mAh·g^−1^ and the remaining capacity was 757 mAh·g^−1^ after 100 cycles. In view of the few works associated with applications of green algae carbon for Li/S batteries, this work provides a new research point for adsorbing soluble polysulfides from biomass resource.
